# Radiation-Induced Airway Distortion Complicating Endotracheal Intubation: A Case Report

**DOI:** 10.7759/cureus.106771

**Published:** 2026-04-10

**Authors:** Abdul R Ansari, Alys Long, Christopher Hadaway

**Affiliations:** 1 Anesthesiology, McLaren Greater Lansing, Lansing, USA; 2 College of Medicine, College of Osteopathic Medicine, Michigan State College of Osteopathic Medicine, East Lansing, USA

**Keywords:** difficult airway assessment, difficult airway management, oropharyngeal neoplasms, radiation-induced fibrosis, endotracheal intubation

## Abstract

Airway management in patients with a history of head and neck radiotherapy presents significant challenges due to radiation-induced fibrosis, anatomic distortion, and friable tissues. These changes increase the risk of difficult mask ventilation, failed intubation, and airway trauma. We present the case of a 77-year-old male with a history of oropharyngeal squamous cell carcinoma treated with chemoradiation who required general anesthesia for jejunostomy tube placement. Preoperative evaluation suggested a potentially difficult airway. Video laryngoscopy revealed a severely distorted and edematous glottic opening surrounded by friable neoplastic tissue, complicated by significant bleeding during intubation. A stepwise airway strategy utilizing video laryngoscopy, aggressive suctioning, and close multidisciplinary coordination resulted in successful endotracheal intubation and an uneventful postoperative course. This case highlights the importance of early airway assessment, careful planning, and the use of advanced airway techniques in patients with prior head and neck radiotherapy.

## Introduction

Patients with a history of head and neck radiotherapy are at increased risk for difficult airway management due to radiation-induced fibrosis, trismus, reduced neck mobility, mucosal edema, and airway narrowing [1]. These changes may progress over time, even years after treatment, and may not be fully appreciated on routine airway examination [2]. Reported rates of difficult intubation in post-radiotherapy patients are significantly higher than in the general population, and failed airway management remains a major cause of anesthesia-related morbidity [3]. Current difficult airway management guidelines from the American Society of Anesthesiologists (ASA) emphasize the importance of thorough preoperative assessment and consideration of advanced airway techniques in patients with anticipated airway difficulty [4]. We report a case of a severely distorted post-radiation airway complicated by friable tissue and bleeding during intubation, emphasizing practical considerations and strategies for safe airway management in this high-risk population.

## Case presentation

**Figure 1:**
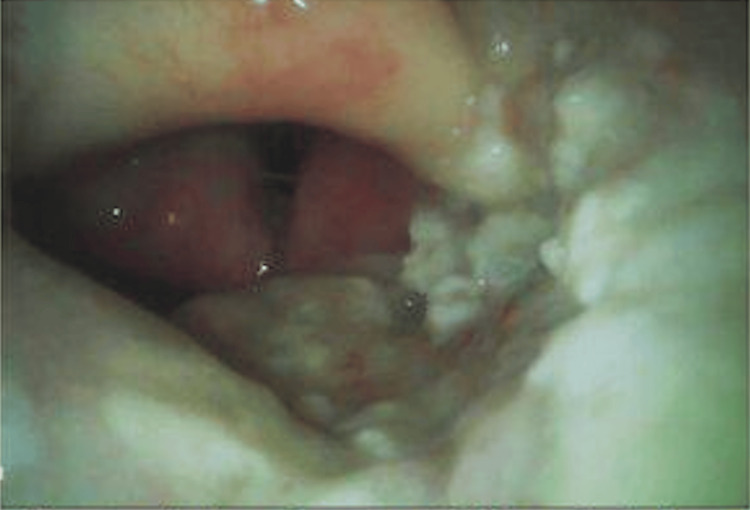
Edematous glottic opening surrounded by neoplastic tissue illustrating distortion of the airway

A 77-year-old male presented for elective jejunostomy tube placement due to progressive dysphagia. His medical history was significant for chronic lymphocytic leukemia, heart failure with reduced ejection fraction, aortic valve replacement, non-Hodgkin’s lymphoma, DVT, and oropharyngeal squamous cell carcinoma treated with radiation. He had multiple prior hospital admissions for pulmonary aspiration and was receiving total parenteral nutrition. Preoperative airway assessment revealed multiple features concerning for a difficult airway, including limited neck range of motion, reduced mandibular mobility with mild trismus, and a Mallampati class III airway. The patient's history of prior head and neck radiation with suspected airway distortion increased the likelihood of fibrosis and decreased tissue compliance. Additionally, his history of recurrent aspiration and progressive dysphagia raised concern for impaired airway protection and potential airway edema. Given these findings, the airway was anticipated to be challenging, and a stepwise airway management plan with advanced airway equipment readily available was formulated. Due to the patient’s aspiration risk and surgical requirements, general anesthesia with endotracheal intubation was planned. After induction of anesthesia with propofol, video-guided intubation was attempted. Video laryngoscopy revealed a markedly edematous and narrowed glottic opening surrounded by friable, irregular tissue consistent with post-radiation changes and recurrent malignancy (Figure 1). Visualization was further compromised by significant bleeding from the oropharyngeal tissue upon manipulation.

**Figure 2:**
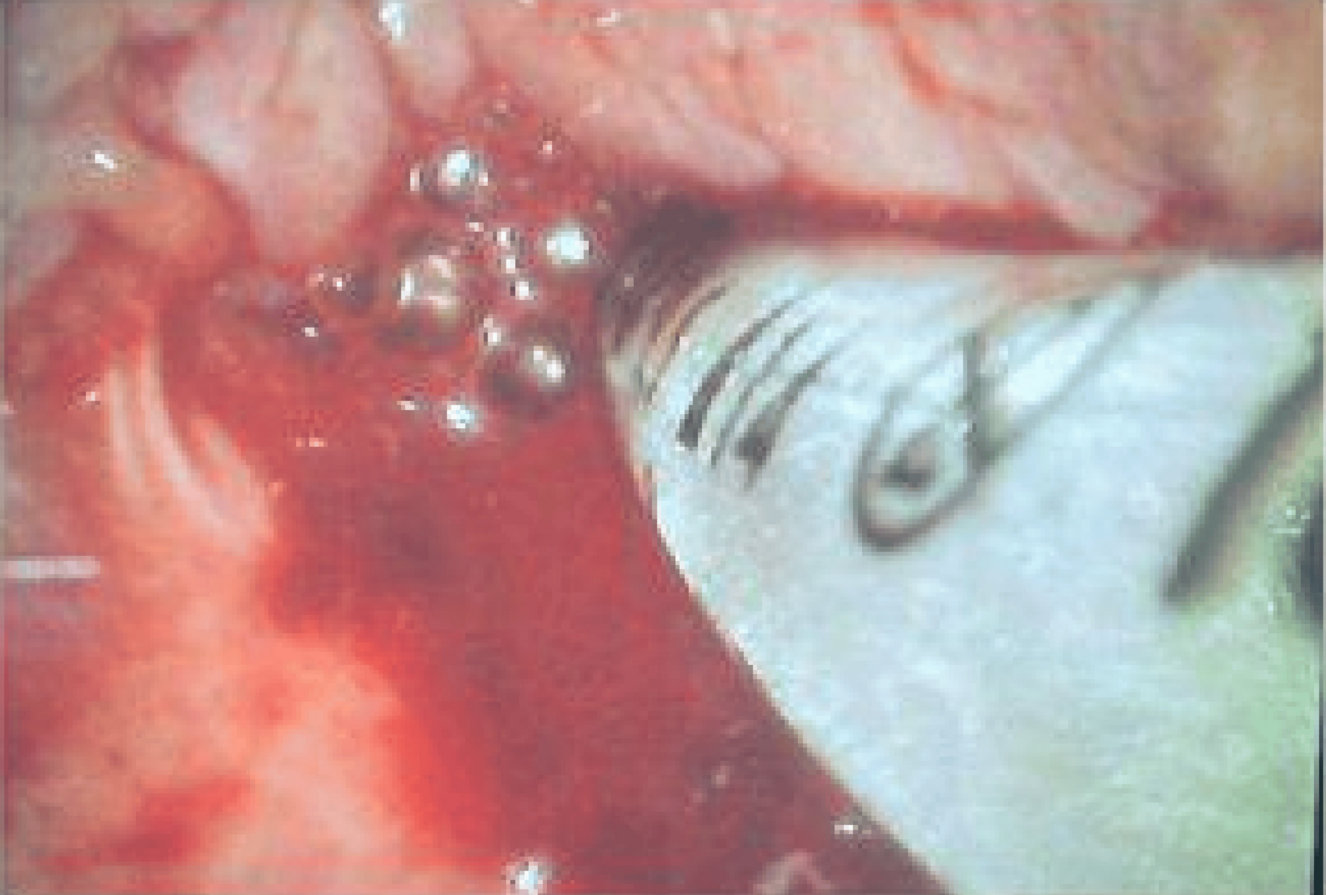
An endotracheal tube was passed through the vocal cords with significant bleeding, highlighting the friability of irradiated tissue

Suction-assisted laryngoscopy and airway decontamination (SALAD) using a Yankauer suction tip were required to maintain visualization. Despite bleeding and distorted anatomy, the endotracheal tube was carefully advanced through the vocal cords under video laryngoscopic guidance (Figure 2). Oxygen saturation remained stable throughout the procedure, and the patient maintained hemodynamic stability without significant fluctuations in blood pressure or heart rate. Repeated oral suctioning was necessary for the remainder of the case due to ongoing oozing from friable tissue. Had video laryngoscopy not been successful, the airway plan included transition to fiberoptic intubation with preservation of spontaneous ventilation, with supraglottic airway placement as a rescue strategy and emergent surgical airway if necessary.

Otolaryngology was notified intraoperatively and recommended topical decongestant therapy to reduce mucosal bleeding. Oxymetazoline was applied prior to extubation. The patient was extubated successfully at the conclusion of the procedure and transferred to the post-anesthesia care unit for close monitoring. No immediate airway complications occurred. Oropharyngeal tissue samples obtained intraoperatively confirmed keratinized squamous cell carcinoma.

## Discussion

Airway management in patients with a history of head and neck radiotherapy presents significant challenges for anesthesiologists due to radiation-induced alterations in airway anatomy and tissue quality. Radiotherapy commonly results in progressive fibrosis, edema, and distortion of normal airway structures [5,6]. These pathophysiologic changes can lead to reduced mouth opening, limited cervical spine mobility, supraglottic narrowing, and impaired alignment of the oral, pharyngeal, and laryngeal axes. As a result, patients with prior head and neck radiation are at substantially increased risk for difficult mask ventilation and tracheal intubation [7]. For this reason, thorough preoperative airway evaluation and preparation are essential in this patient population.

In the present case, a patient with a history of oropharyngeal squamous cell carcinoma treated with radiotherapy demonstrated significant airway distortion and friable mucosal tissue during laryngoscopy. Radiation-associated mucosal fragility increases the likelihood of bleeding with even minimal airway manipulation. In this case, blood within the airway obscured visualization of the glottic structures and required repeated suctioning to maintain an adequate view. Similar findings have been reported in patients with prior head and neck radiation, where vascular fragility and tissue edema contribute to increased bleeding risk during airway instrumentation [8]. These factors may complicate traditional direct laryngoscopy and increase the likelihood of failed intubation attempts if alternative airway strategies are not considered [9].

Video laryngoscopy proved to be a valuable tool in achieving successful intubation in this case. In addition, flexible fiberoptic bronchoscopy remains an important adjunct for airway management in this population and may be considered when significant airway narrowing or anticipated difficulty with conventional techniques exists. The availability of advanced airway devices and a clearly defined stepwise airway plan are, therefore, critical when managing patients with prior radiation exposure [10,11].

This case also emphasizes the importance of reassessing airway anatomy in patients with prior head and neck radiation who require repeated anesthetic encounters. Radiation-induced changes may progress over time, meaning that patients who previously underwent uncomplicated intubation may subsequently develop difficult airway characteristics [1]. Continuous reassessment and individualized airway planning are therefore necessary for each anesthetic exposure. For this reason, a scheduled preoperative evaluation in the days leading up to surgery can further facilitate thorough airway assessment and multidisciplinary planning for patients with known or anticipated difficult airways.

Overall, this case highlights the complex airway considerations associated with prior head and neck radiotherapy. Early recognition of radiation-related airway changes, combined with careful preoperative assessment, use of advanced airway visualization techniques, and multidisciplinary collaboration, can significantly improve the likelihood of successful airway management and reduce perioperative morbidity in this high-risk patient population.

## Conclusions

Airway management in patients with a history of head and neck radiotherapy requires meticulous preoperative assessment, careful planning, and the use of advanced airway techniques. Video laryngoscopy and proactive suctioning can facilitate successful intubation in the presence of distorted anatomy and bleeding. Early recognition of radiation-induced airway changes and a multidisciplinary approach are essential to minimizing perioperative complications and improving patient outcomes.
